# The Search for Disease Modification in Parkinson’s Disease—A Review of the Literature

**DOI:** 10.3390/life15081169

**Published:** 2025-07-23

**Authors:** Daniel Barber, Tissa Wijeratne, Lakshman Singh, Kevin Barnham, Colin L. Masters

**Affiliations:** 1 Department of Neurology, Sunshine Hospital, Western Health, St Albans, VIC 3021, Australia; daniel.barber@wh.org.au (D.B.); lakshman.singh@vu.edu.au (L.S.); 2 Florey Institute of Neuroscience and Mental Health, University of Melbourne, Parkville, VIC 3052, Australia; kevin.barnham@florey.edu.au (K.B.); c.masters@unimelb.edu.au (C.L.M.); 3 Australian Institute of Migraine, 522, Bell Street, Pascoe Vale South, VIC 3044, Australia; 4 Migraine Foundation Australia, 47, Milleara Road, Keilar East, VIC 3033, Australia; 5 Department of Public Health and Psychology, La Trobe University, Bundoora, VIC 3086, Australia; 6 Department of Medicine, University of Rajarata, Saliyapura 50008, Sri Lanka; 7 College of Health and Biomedicine, Victoria University, Melbourne, VIC 3021, Australia

**Keywords:** Parkinson’s disease, oxidative stress, mitochondria, disease modification

## Abstract

Sporadic Parkinson’s Disease (PD) affects 3% of people over 65 years of age. People are living longer, thanks in large part to improvements in global health technology and health access for non-neurological diseases. Consequently, neurological diseases of senescence, such as PD, are representing an ever-increasing share of global disease burden. There is an intensifying research focus on the processes that underlie these conditions in the hope that neurological decay may be arrested at the earliest time point. The concept of neuronal death linked to ageing- neural senescence- first emerged in the 1800s. By the late 20th century, it was recognized that neurodegeneration was common to all ageing human brains, but in most cases, this process did not lead to clinical disease during life. Conditions such as PD are the result of accelerated neurodegeneration in particular brain foci. In the case of PD, degeneration of the substantia nigra pars compacta (SNpc) is especially implicated. Why neural degeneration accelerates in these particular regions remains a point of contention, though current evidence implicates a complex interplay between a vast array of neuronal cell functions, bioenergetic failure, and a dysfunctional brain immunological response. Their complexity is a considerable barrier to disease modification trials, which seek to intercept these maladaptive cell processes. This paper reviews current evidence in the domain of neurodegeneration in Parkinson’s disease, focusing on alpha-synuclein accumulation and deposition and the role of oxidative stress and inflammation in progressive brain changes. Recent approaches to disease modification are discussed, including the prevention or reversal of alpha-synuclein accumulation and deposition, modification of oxidative stress, alteration of maladaptive innate immune processes and reactive cascades, and regeneration of lost neurons using stem cells and growth factors. The limitations of past research methodologies are interrogated, including the difficulty of recruiting patients in the clinically quiescent prodromal phase of sporadic Parkinson’s disease. Recommendations are provided for future studies seeking to identify novel therapeutics with disease-modifying properties.

## 1. Parkinson’s Disease—Epidemiology, Aetiology, Current Treatments and the Search for Disease Modification

Parkinson’s disease is the second most common neurodegenerative disease worldwide and is exceeded only by Alzheimer’s dementia. As global populations age, many clinicians are predicting an impending ‘Parkinson pandemic,’ with the incidence of Parkinson’s Disease predicted to double in 20 years and triple by 2050 [1,2]. Over 90% of PD cases are thought to arise because of a complex interaction between a patient’s genetic profile across numerous loci and unidentified environmental factors (sporadic PD). Recent genome-wide association studies have identified over 90 independent risk variants for PD [3]. The remaining 5–10% of cases have been linked to single gene mutations in LRRK2, SNCA, PARKIN, PINK-1, MAPT, VPS35, and GBA genes. Clinical manifestations and histopathological findings vary considerably between these mono-allelic forms and likely represent distinct pathophysiological mechanisms from the more prevalent sporadic form [4]. Nevertheless, each of these mutations leads to selective destruction of dopaminergic neurons and provides invaluable insights into the mechanisms driving neural degeneration in the more common sporadic form.

The classic motor features of Parkinson’s Disease- bradykinesia, rigidity, and tremor—are a consequence of degeneration within dopaminergic systems within the midbrain and basal ganglia [5]. By the time motor features are manifested anywhere between 40 and 60% of dopaminergic neurons within the substantia nigra pars compacta have already degenerated [6]. Historically, there has been little focus on the ‘non-motor’ features of Parkinson’s disease, which may predate the emergence of motor symptoms by decades. These non-motor features include anosmia, constipation, autonomic dysfunction, and sleep disorders such as REM-sleep behaviour disorder. Subclinical changes in visual processing, cognitive dexterity, and mood are now identified in patients with prodromal PD [7,8]. As the disease progresses, cognitive features including cognitive impairment or dementia as well as hallucinations, personality change, and executive dysfunction become increasingly disabling and greatly contribute to morbidity [9].

Currently available treatments for PD act principally on motor dysfunction by upregulating dopamine within the midbrain and basal ganglia [10]. Premetabolites of dopamine (levodopa) and synthetic dopamine agonists remain the mainstay of symptomatic treatment, but can modify disease outcome only indirectly—by optimizing patient function, thereby supporting functional strength and condition treatment [10], but unfortunately, these agents have not been demonstrated to modify neurological decay [11]. Consequently, patients gradually lose responsiveness to dopamine therapy, necessitating increasing doses, which in turn result in dopamine-related side effects such as dyskinesia, autonomic dysfunction, impulsivity, and psychosis [12,13].

Disease-modifying therapy represents the elusive “Holy Grail” in the treatment of neurodegenerative diseases such as PD. Disease modification pertains to therapies that could slow disease progression or promote neural recovery or compensation, thereby preventing or reversing the disease mechanism rather than relying on crude dopamine-based therapies [14]. Over the last 30 years, hundreds of novel therapeutics have been evaluated as potential disease-modifying agents for PD. Unfortunately, while many have shown promise in animal models and ex vivo studies, none have demonstrated the ability to arrest or slow the progression of PD in humans [15]. This article reviews these trials and discusses contemporary approaches in the search for disease-modifying agents in PD.

## 2. Methodology

**Figure 1:**
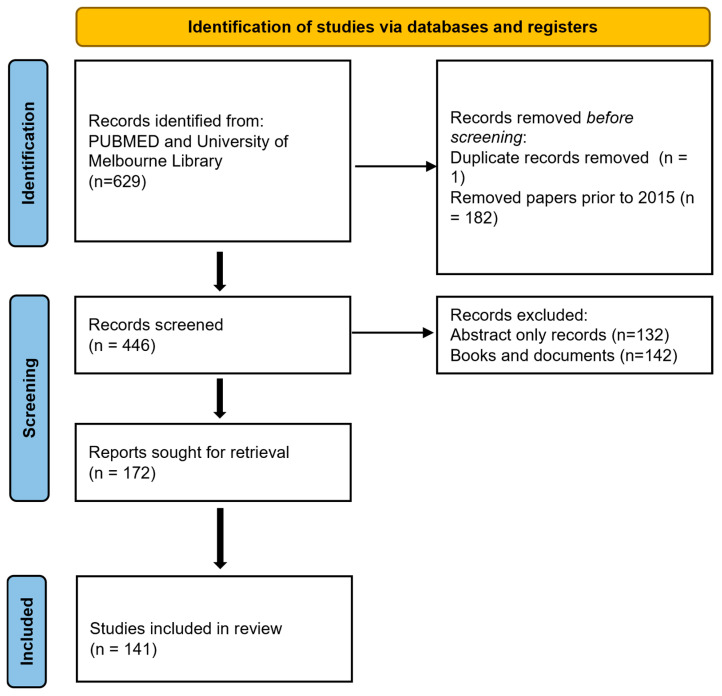
PRISMA chart.

LS and DB independently conducted a PubMed search on 15 October 2024 using the terms: Parkinson’s Disease AND disease modification AND neurodegeneration. Articles published before 2015 were excluded unless they were essential to the current understanding. Relevant review and summary articles were included. Additional key references cited within these papers, as well as important studies not captured by the original search terms, were also reviewed and incorporated into the discussion (see Figure 1). TW, KB, and CM reviewed and resolved any conflicting interpretations, ensuring consensus on the final selection and analysis of the literature.

## 3. Neuropathophysiology of Parkinson’s Disease—What We Know

### 3.1. Alpha-Synuclein Aggregates, Known as Lewy Bodies, Are the Histopathological Hallmark of PD

Parkinson’s Disease is caused by a stereotyped pattern of neurodegeneration across multiple brain regions. Intracytoplasmic aggregates of fibrillar alpha-synuclein (aS), called lewy bodies, are the histopathological hallmark of Parkinson’s disease and the closely related lewy body dementias (DLB) [16]. Early studies point towards a close relationship between CNS protein status and clinical outcome, with the ratio of fibrillar aS to Abeta1-42 in CSF not only predicting the development of sporadic PD and DLB but also indicating which patients are prone to motoric symptoms and which will develop cognitive impairment.

**Figure 2:**
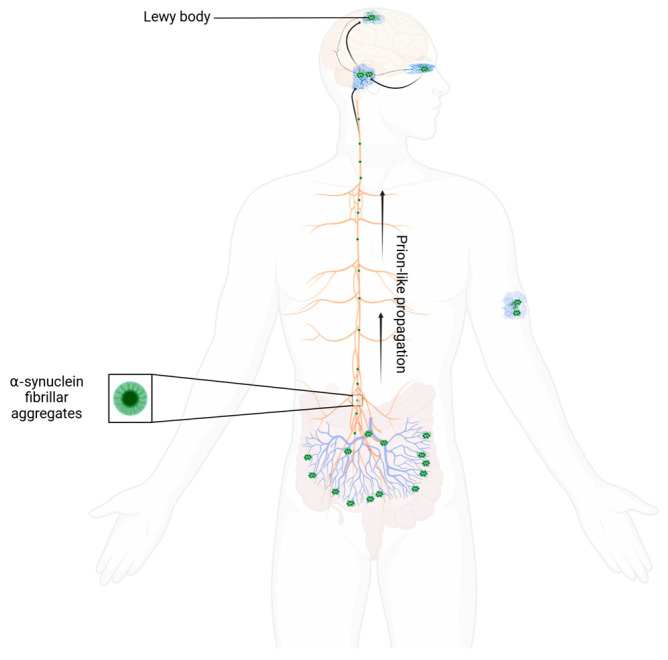
The Braak Hypothesis for aS propagation within the peripheral and central nervous system. Arrows indicating the proposed pathway of lewy body changes, from peripheral centres, including the enteric plexus and olfactory nucleus, to central regions, including the brainstem, midbrain, and higher cortical regions. This diagram also demonstrates how lewy bodies have been demonstrated in skin tissue.

Braak and colleagues first described fibrillar aS deposition spreading in a caudal-rostral pattern [17]. They hypothesized that the pattern of neurodegeneration in PD followed this course of lewy body spread (Figure 2). In their model, the pathological process of sporadic PD begins with the aggregation of lewy bodies within two peripheral nerve sites, namely the enteric plexus and olfactory nerve. This process advances to lower brain regions over time, including the dorsal motor nucleus, reticular formation, and olfactory nucleus (Braak stages 1 and 2). These early stages of the disease may correspond to the emergence of early premotor features, including autonomic dysfunction, anosmia, and REM sleep behavior disorder (RBD). Over time aS deposition spreads to the rostral midbrain and basal ganglia, infiltrating the dopaminergic pathways within the substantia nigra and ventral tegmental area (Braak stages 3 and 4). The neurodegeneration that accompanies aS deposition in this region is insidious and initially silent, with PET studies demonstrating neurodegeneration occurring many years before the onset of motor symptoms [18,19]. In the more advanced stages of PD (Braak stages 5 and 6), aS deposits can be detected in higher cortical regions, notably in the mesial temporal lobe, primary motor and sensory cortices, and prefrontal regions. It is hypothesized that this stage corresponds to the emergence of higher-order cognitive changes in memory, visuospatial processing, and executive function. This apparent chain-like spread of Lewy bodies could indicate that abnormal aS aggregation is a self-propagating process akin to a prion disease [20]. Indeed, preliminary cell studies have demonstrated this prion-like behaviour of aS and its ability to self-propagate between neighbouring neurons [21].

**Figure 3:**
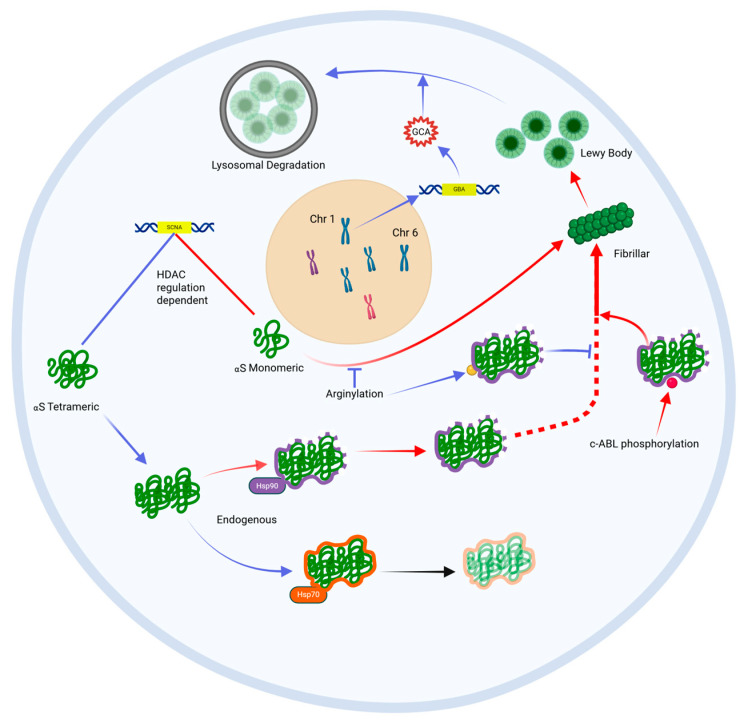
HDAC regulators control SCNA expression of either monomeric or tetrameric alpha-synuclein (aS). Monomeric aS is prone to fibrillar confirmational change. Tetrameric aS is protected from confirmational change by the chaperone protein, Hsp70. c-abl mediated phosphorylation on non-Hsp70-chaperoned tetrameric aS drivers fibrillar confirmational change of tetrameric aS. Arginylation of aS inhibits fibrillar confirmational change. Fibrillar aS aggregates to form lewy bodies. Lysosomal degradation is an important mechanism of lewy body eradication. Blue = protective pathway; Red = pathological pathway.

Neurons incorporate several mechanisms to prevent the accumulation of pathological aS, a process referred to as ‘proteostasis’ (Figure 3) [22]. Failure of these mechanisms may trigger and/or exacerbate this accumulation of aS as Lewy bodies. One such mechanism is epigenetic regulation through histone acetylation of DNA chromatin [23]. A study by Park et al. demonstrated changes in histone acetylation (HATs) and deacetylation enzymes (HDACs) involved in the regulation of the alpha-synuclein gene (SNCA) that were unique to the SNpc of Parkinson’s patients, suggesting a potential dysregulation of gene transcription underlying the accumulation of aS in this cohort [24]. Other studies have highlighted the importance of the relative activity of two competing chaperone proteins, Hsp70 and Hsp90, which influence cellular management of aS [25]. More specifically, Hsp70 appears to protect against lewy body aggregation by binding to endogenous aS thereby preventing its accumulation and promoting lewy body disassembly, whereas Hsp90 does not provide this protective function and thereby promotes lewy body aggregation [26]. Other proteins, called HSF-1 modulators, regulate the activity of these Hsp chaperone proteins and may influence aS proteostasis in this way. The most common gene associated with early-onset PD, GBA, encodes a lysosomal enzyme glucocerebrosidase (GCA). GCA is critical for lysosome-mediated sequestration and degradation of aS, and loss-of-function mutations in the GBA gene lead to the accumulation of aS in affected carriers [27,28]. A knock-in GBA1 mouse model has demonstrated that increased GCA expression may also regulate SCNA expression of aS from its aggregation-prone monomeric form to a benign tetrameric form that is less prone to Lewy body aggregation, and this cellular function appears protective against motoric parkinsonism [29].

Post-translational modification of aS can alter its propensity to aggregate into pathological Lewy bodies. Arginylation (the addition of the amino acid arginine to the N-terminus of amino acids) within the peptide chain can reduce aS aggregation [30]. On the other hand, c-abl enzyme-driven phosphorylation of tyrosine 39 of the aS peptide leads to abnormal protein folding and aggregation. This enzyme is indeed overexpressed in diseased neurons in PD [31]. Phosphorylation of serine 129 (S129) is the dominant post-translational modification found in Lewy-body-aggregated aS, present in up to 90% of Lewy bodies, compared with 4% in healthy samples [32,33,34].

In addition to Lewy bodies, up to 50% of patients with PD eventually develop a sufficient burden of beta-amyloid plaques as well as tau-containing neurofibrillary tangles to justify a second diagnosis of Alzheimer’s dementia [35]. Individuals with AD-PD overlap are pathologically distinct from the pure Parkinson’s Disease Dementia (PDD) cohort, with a recent antibody study indicating different tau species expressed between the two groups [36]. This suggests that while aS is central to the pathological process of PD, synergistic accumulation of different pathological protein bodies may precipitate more rapid neurological and cognitive decline in some sufferers and could have real-world implications for disease-modifying therapies.

While lewy bodies are the histopathological hallmark of idiopathic Parkinson’s disease, their precise role in neural degeneration remains unclear. While it is possible that Lewy bodies, or the fibrillar aS peptides that form them, cause neural degeneration, they may also represent a biochemical byproduct of another fundamental cell death pathway.

### 3.2. Oxidative Stress and Mitochondrial Dysfunction Induce Innate Immune Processes That Drive Cell Death in PD

**Figure 4:**
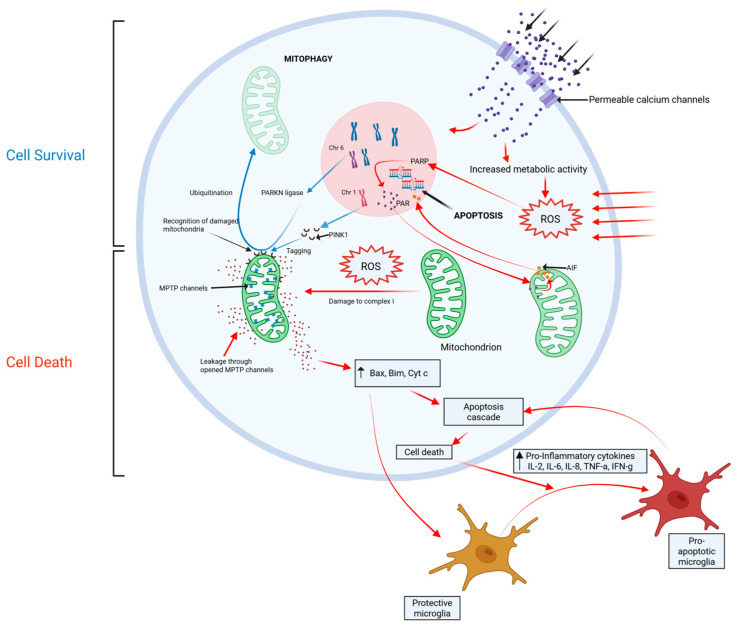
High intracellular metabolic activity and extracellular stressors drive the creation of reactive oxygen species (ROS). ROS upregulate PARP and its product PAR, which triggers apoptosis through the release of apoptosis-inducing factor (AIF) from the mitochondrial membrane. ROS also causes damage to complex 1 of the electron transport chain, leading to the release of further pro-apoptotic species from the mitochondrial membrane (bax, bim, cytC). Cell apoptosis induces pro-inflammatory cytokines, which drive the conversion of protective microglia to pro-apoptotic microglia, perpetuating cell apoptosis. Mitophagy protects against apoptosis through the breakdown of diseased mitochondria. This process is mediated by PINK1, which tags diseased mitochondria for ubiquitination by PARKN ligase. Blue = protective pathway; Red = Pathological Process.

The innate immune response (Figure 4), triggered by oxidative stress, is fundamental to disease progression [37]. Degenerating neurons in the substantia nigra are highly complex, unmyelinated fibers with an unusually high metabolic demand. SNpc neurons also have pacemaker Ca^2+^ channels that regulate their background activity. It is likely that these properties make these cell types particularly vulnerable to the production of reactive oxygen species and their harmful downstream effects. Oxidative stress is harmful to mitochondria, causing dysfunction in complex 1 of the electron transport chain [38]. Diseased mitochondria can trigger apoptosis through processes such as abnormal activation of cytochrome C, upregulation of pro-apoptotic proteins Bax and Bim, and opening of mtPTP channels within the mitochondrial membrane, which trigger pro-apoptotic cascades within the cell cytoplasm (Figure 4) [39]. Parthanatos, another mitochondria-mediated mechanism of cell death, may also be triggered when reactive oxygen species upregulate poly (ADP-ribose) polymerase (PARP-1) activity, which in turn triggers the release of apoptosis-inducing factor (AIF1) from the mitochondrial membrane [40]. The removal of damaged mitochondria is fundamental to protecting neurons against downstream cellular processes that lead to apoptosis. Mutations in the PARKN gene can cause early-onset PD. PARKN encodes a ubiquitin ligase that drives the adaptive breakdown of dysfunctional mitochondria, a protective process called ‘mitophagy’. The loss of this important cell function leads to neuronal death and results in Parkinsonism in late childhood or early adulthood [41]. A related gene mutation in PINK1 is another genetic cause of early-onset PD. The PINK1 protein tags pathological mitochondria for recognition by PARKN, lending further support to the notion that pathological mitochondria damaged by reactive oxygen species are central to neuron death.

LRRK2 encodes a protein involved in diverse protective cellular functions, including management of oxidative stress, inflammatory cascades, protein stabilization, mitochondrial stabilization, and mitophagy. A mutation within the kinase segment of this gene- the G2019S mutation- is the most common cause of autosomal dominant Parkinson’s disease, providing even further evidence of the centrality of these cell functions in the genesis of PD [42].

Microglia, the resident immune cells in the central nervous system, can modify the downstream cellular effects of oxidative stress and mitochondrial dysfunction, and determine the innate immune response against affected neurons. In non-PD brain tissue, protective microglia suppress the immune attack of dopaminergic neurons through neuron-glia crosstalk, histone modification, and mRNA regulation [43]. The transition to pro-apoptotic microglia through an unidentified signaling mechanism may promote cell death in response to oxidative stress and mitochondrial dysfunction. Supporting the role of the immune response in the genesis of PD, studies have demonstrated elevated serum levels of inflammatory cytokines such as IL-2, IL-6, IL-8, TNF-α, and IFN-γ in PD sufferers [44,45]. PD sufferers also demonstrate a lower systemic lymphocyte count and higher neutrophil-to-lymphocyte ratio (NLR) than healthy controls [46]. More recently, there has been an increasing focus on the role of the signaling molecule sphingosine-1-phosphate (S1P) in attenuating microglial-driven neuronal apoptosis [47]. An association study of 392 participants demonstrated under-expression of S1P in the serum of PD sufferers compared with matched controls. In 64 PD participants who were followed prospectively, advancing PD severity was correlated with a progressive reduction in serum S1P levels [48].

### 3.3. Reciprocal Reinforcement of Oxidative Stress and Alpha-Synuclein Aggregation

**Figure 5:**
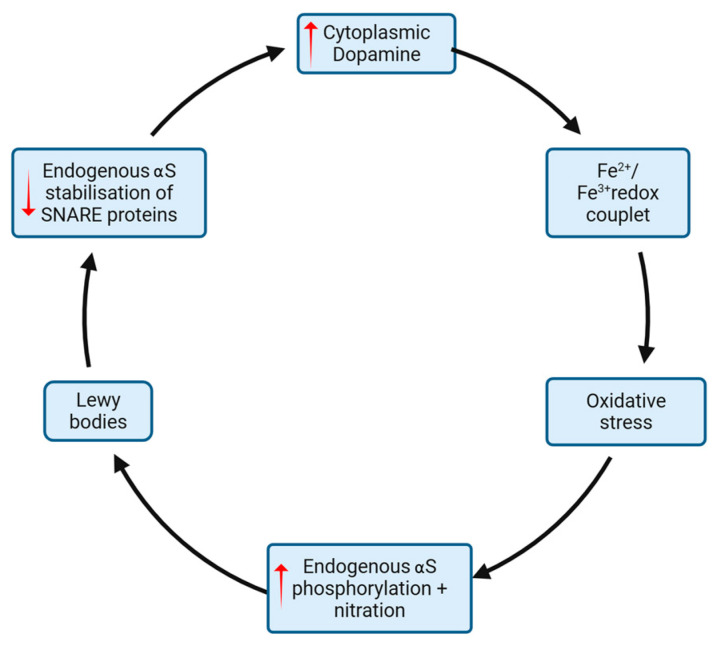
Lewy body formation and oxidative stress (and their downstream pro-apoptotic cascades) are mutually reinforcing. Lewy body aggregation leads to a reduction in endogenous tetrameric aS, which reduces SNARE protein stabilization of dopamine-containing endosomes. This leads to an increase in free cytoplasmic dopamine, which causes oxidative stress through the creation of an unstable Fe^2+^/Fe^3+^ redox coulet. This oxidative stress drives phosphorylation and nitration of aS, leading to the formation of the fibrillar species, which aggregate to form more lewy bodies. Red = pathological change.

As well as being a driver of mitochondrial injury and inflammation, it has been demonstrated that oxidative stress can trigger the pathological phosphorylation and nitration of tyrosine residues within aS oligomers, which leads to the formation of lewy bodies [49]. In addition, model studies suggest that excessive Ca^2+^ influx—a known cause of oxidative stress—is a key driver of overproduction of s129 aS, the aS form most associated with lewy body deposition as mentioned above. In other words, oxidative stress may directly drive the aggregation of aS as lewy bodies in diseased neurons (Figure 5).

While pathological alpha-synuclein deposits may indeed be caused by oxidative stress, some evidence also suggests the opposite. That is, misfolded aS accumulation may in turn drive oxidative stress. A central function of endogenous aS is the stabilization of SNARE proteins, which aggregate dopamine in presynaptic vesicles and allow vesicular fusion with the presynaptic endplate for its release into the synapse. Therefore, loss of this function may lead to the accumulation of cytoplasmic dopamine, a recognized generator of oxidative stress, within the cell cytoplasm (Figure 5). The mechanism by which cytoplasmic dopamine induces oxidative stress is likely multifactorial, although a key mechanism involves the formation of an unstable redox couplet with ferrous/ferric cations (Fe^2+^/Fe^3+^) [50]. This relationship between Fe^2+^/Fe^3+^ and cytoplasmic dopamine is made all the more compelling by imaging studies demonstrating that the PD brain undergoes disproportionate iron deposition in areas of known degeneration where dopamine stores are highest, namely the SNpc [51]. The propagation of pathological aS may also lead to oxidative stress through the loss of other protective properties of functioning aS. For example, endogenous aS may play a role in sequestering early peroxidation products of fatty acids. On the other hand, fibrillar aS—the form associated with lewy bodies—demonstrates an 80% reduction in this scavenging property, which increases cell exposure to these reactive fatty acid metabolites [52]. To summarise, the cell processes underlying neuronal death in sporadic PD may involve a complex reciprocal interaction between oxidative stress, cell pathways, and the accumulation of pathological proteins such as aS (Figure 5).

## 4. Targets for Disease Modification (See Table 1)

These mechanisms of neural degeneration present a large array of targets for disease modification. Prior studies have focused on four primary strategies: (1) attenuation, prevention, or reversal of alpha-synuclein deposition, (2) modification of oxidative stress and immune responses leading to cell death, (3) regeneration or replacement of lost neurons and (4) repurposing old drugs hypothesized to have disease modifying properties. Table 1 and Table 2 at the end of this section provide an overview of the studies discussed, categorized into preclinical studies (animal modes, ex vivo studies and population association studies) (Table 1) and clinical trials (Table 2).

### 4.1. Prevention and Attenuation of Alpha-Synuclein Aggregation

Treatments that prevent or reverse the accumulation of aS and formation of lewy bodies could provide a pathway to disease modification.

Any intervention that suppresses the expression of SCNA, the gene encoding aS, could theoretically prevent aS aggregation. Stimulation of the beta-2 receptor may confer this very advantage [53]. A large cohort study in Norway demonstrated that patients who regularly received salbutamol, a sympathomimetic agent that acts via selective beta-2 adrenergic receptor stimulation, had a reduced risk of developing PD compared to the general population [54]. In contrast, patients who used propranolol, a beta receptor antagonist, demonstrated a modest increase in the relative risk of PD. This link is only an association, however, and critics point out the possibility of reverse causation because prodromal PD patients are more likely to present with non-specific action tremors, which may in fact herald the onset of PD, and that are commonly treated with propranolol [55].

One fundamental way SCNA transcription can be attenuated is by targeting the specific epigenetic processes that control gene transcription. Mazzocchi et al. suggested that class 2 histone deacetylases (HDACs) represent compelling targets for epigenetic suppression of SCNA expression. Preliminary studies indicate that modification of the upstream SCNA gene regulators, namely FTO, can achieve a similar effect on aS expression. Introduction of m6A demethylase FTO (siFTO) to neurons via mesenchymal stem cell-derived exosomes has been shown to slow dopaminergic neuron death in PD mouse models [56].

As previously discussed, GCA is an enzyme involved in the lysosomal sequestration of misformed aS. It also modifies SCNA gene expression to transcribe a benign form of aS with a low propensity for aggregation. A phase 1 trial of LTI-291, an allosteric activator of GCA, has demonstrated good tolerability among 40 GBA-PD sufferers, with a long-term RCT to follow [57].

As highlighted in Braak’s hypothesis discussed above, aS deposition is known to first accumulate in the peripheral nervous system, particularly the enteric plexus innervating the gastrointestinal tract. The gut microbiota is a complex ecosystem of bacteria that is in constant interface with enteric neurons and has been the focus of PD research for many years. PD is associated with higher gut levels of verrucomicrobiaceae (gram-negative, obligate anaerobes) and lower levels of prevotellaceae (gram-negative anaerobes) compared with healthy controls [58]. The relative concentrations of these organisms affect toll-like receptor (TLR) expression in gut dendritic cells, which impacts mucosal permeability to aS, influencing its aggregation and propagation throughout the body. In MPTP mice models, fecal micro-transplantation can reconstitute a protective gut microbiota profile and has been shown to protect against striatal neuron loss [59].

Other studies have focused on altering the post-translational modifications, aggregation, and degradation of aS. UCB0599 is a novel small-molecule inhibitor of aS misfolding and aggregation, which demonstrates good brain penetrance. Phase 1 trials have suggested good tolerability in human subjects [60]. ABT-888, a small molecule inhibitor of PARP-1 designed to dampen parthanatos-mediated cell death, has been shown to, in fact, prevent misfolding of aS into its aggregation-prone fibrillar form, demonstrating the intimate relationship between these pathological cell processes [40]. Several studies have explored the manipulation of endogenous chaperones, or heat shock proteins, which can prevent the aggregation of aS by facilitating its ubiquitination and upregulating proteosomal activity within cells (see above) [61]. Geldanamycin is one such agent that inhibits Hsp90. Pretreatment with geldanamycin protects against dopaminergic neurodegeneration in MPTP mouse models of Parkinson’s Disease [62]. Other novel therapeutics targeting the upregulation of the protective Hsp70 demonstrate a similar effect in these mouse models. FTY720 (fingolimod) is another enzyme involved in aS degradation, and one study has demonstrated the practicality of using a chitosan nanocarrier to introduce FTY720 into neurons [63]. A nanoscavenger molecule (NanoCA) delivered nasally has been shown to upregulate exosomal clearance of aS in mouse models by upregulating transcription factor EB, a major autophagy regulator, thereby protecting against MPP^+^-induced dopaminergic toxicity [64] Iron sequestration has been theorized as a mechanism of neuroprotection in Parkinson’s Disease, by interrupting the interaction between aS deposition and potentially toxic iron accumulation. A phase one study of PBT434, a small molecule with a partial affinity iron-binding motif, reduced iron load and iron-mediated aS aggregation in 6-OHDA, MPTP, and transgenic PD mouse models, and resulted in the rescue of motor function [65]. Disappointingly, a recent human study of another iron chelator, deferiprone, demonstrated no clinical benefit over a 36-week treatment period, with a signal towards harm with more participants in the treatment arm progressing to increased levodopa requirements [66]. GYY4137, a novel therapeutic agent, has been shown to prevent aS nitration in mouse models by releasing hydrogen sulphide (H_2_S), which sequesters reactive oxygen species. However, no study demonstrates its efficacy in humans [67]. As previously mentioned, the tyrosine kinase c-abl phosphorylates aS and potentiates its misfolding and aggregation. Additionally, it appears to inhibit the neuroprotective function of PARKN. c-abl inhibitors such as nilotinib have shown neuroprotective properties in animal models of PD [68]. Unfortunately, multiple human trials on nilotinib have failed to demonstrate any meaningful clinical benefit over 12-month treatment periods [69,70]. Nilotinib poorly traverses the blood-brain barrier, and many researchers still believe an alternative c-abl inhibitor with good CSF bioavailability represents a promising therapeutic pathway [71]. One such candidate c-abl inhibitor, vodobatinib, has so far demonstrated reasonable tolerability in phase 1 human trials [72].

Immunotherapies are the most advanced and targeted therapies to accelerate the removal of pathological aS. An Austrian phase 1 trial demonstrated the safety of PDO1A, a novel 8-amino acid-peptide that mimics an epitope of the C-terminus of aS and induces active immunity against endogenous aS [73]. Doses between 15 and 75 mg showed good tolerability and triggered an active humoral response in PD patients. Those who received the 75 mg dose showed reduced CSF concentrations of oligomeric aS. Passive immunotherapies—exogenously produced antibodies against aS—have shown early promise. Hung et al., in their review ‘Approaches to Disease Modification for Parkinson’s Disease: Clinical Trials and Lessons Learned’, provide an exhaustive list of passive immunotherapies currently in early phase trials, some of which have demonstrated good tolerability. These agents include prasinezumab (PRX002/RG7935), a humanized monoclonal antibody against the C-terminus of aS. This agent demonstrated good tolerability in a phase 1 trial. A recent phase 2 trial did not reach its primary endpoint of improvement in clinical status among participants, although an extended 2b trial has demonstrated modest attenuation of motor decline as measured by the MDS-UPDRS part 3 scale [74,75]. More recent work by the Pagano group did not demonstrate a benefit to prasinezumab, with a phase 2 clinical trial over 1 year failing to meet its primary or secondary clinical endpoints, and SPECT imaging not demonstrating protection of dopamine transporter density in the putamen (PASADENA trial) [76]. Similarly, cinpanemab, another monoclonal antibody to aS, showed no clinical benefit over a 52-week treatment period [77].

### 4.2. Modification of Oxidative Stress, Mitochondrial Dysfunction, and Neuroinflammation

Other studies have focused on modifying oxidative stress, mitochondrial dysfunction, and the resultant immune responses, which can lead to neuronal death. Fat-soluble vitamins are a valuable source of antioxidants and have been considered for disease modification for decades. Vitamin E, vitamin C, and polyphenols interact with reactive oxygen species and terminate oxidative chain reactions [39]. Multiple studies have demonstrated that higher dietary vitamin E intake correlates inversely with the risk of PD [78]. The first clinical trial using vitamin E for treating early symptomatic PD was carried out over 25 years ago (DATATOP study), and it failed to demonstrate a sustained clinical benefit [79]. A randomized controlled trial in 2017 demonstrated a modest but statistically significant improvement in UPDRS scores after 12 weeks of treatment with high-dose vitamin E and omega-3 fatty acids [80]. Further studies that incorporate a clinical washout period are needed to investigate the long-term effects of high-dose vitamin E supplementation on PD progression. However, many researchers doubt the clinical utility of vitamin E because it poorly traverses the blood-brain barrier. N-acetyl cysteine (NAC) is a precursor of glutathione, a powerful antioxidant. Many studies have explored the potential of NAC to dampen reactive oxygen species and inflammation in neurodegenerative diseases [81]. NAC administration in 6-OHDA PD mouse models proved to be neuroprotective [82]. A trial of 42 patients with IPD demonstrated that combined intravenous infusion and oral supplementation of NAC led to increased dopamine transporter (DAT) binding in the caudate and putamen [83]. Unfortunately, intranasal glutathione treatment did not alter the clinical outcomes of PD participants [84]. Urate, a byproduct of purine metabolism, is another powerful endogenous antioxidant that activates the Nrf2 antioxidant pathway. In 2017, Crotty et al. demonstrated that treatment with purine inosine could elevate CSF urate concentrations and was well-tolerated in PD participants [85]. Unfortunately, a 2-year trial of inosine in 298 participants with PD failed to meet its primary endpoint of slowing UPDRS clinical disease progression [86]. Celastrol is another potent antioxidant agent that also activates the Nrf2 pathway and has shown disease-modifying effects in the MPTP PD mouse model [87]. Other novel scavenger antioxidants are currently under investigation for their neuroprotective effects, including two scavenger antioxidants (lipophilic metalloporphyrins), which have been shown to preserve ventral midbrain dopaminergic neurons in 6-OHDA Parkinson’s mouse models [88].

Removal of diseased mitochondria can circumvent downstream cell death pathways. Rapamycin, as well as coenzyme Q10, was shown to attenuate mitochondrial dysfunction in stem cells derived from PINK-1 and LRRK2 mutation carriers [89]. A study from 2020 indicates that activation of mu-opioid receptors can protect human PC12 cells against mitochondrial dysfunction by upregulating PARKN-mediated mitophagy [90]. This may prove significant as hydrocortisone, the ubiquitous anti-inflammatory steroid, has been shown to increase PARKN levels in the 6-OHDA mouse model, leading to increased dopaminergic neuron survival [91].

Targeted anti-immune treatments can circumvent the common pathway by which oxidative stress and mitochondrial dysfunction lead to neuronal death. Long-term caffeine intake is negatively correlated with the risk of PD. It has been postulated that caffeine may confer this advantage by preventing microglial activation via the inhibition of adenosine A2A receptors [92]. Intriguingly, activation of the endocannabinoid system through the use of targeted THC and CBD phytocannabinoids may confer a similar advantage by redirecting M1 microglia to the neuroprotective M2 phenotype [93]. Novel ligands that modify microglial reactivity, such as the nuclear receptor-4A2 ligand C-DIM12, have demonstrated a neuroprotective effect in MPTP knockout mice, whereas a liposome vector that delivers dexamethasone directly to CD163^+^ resident macrophages in the basal ganglia can protect dopaminergic neurons in the 6-OHDA Parkinson’s mouse model [94,95] Finally, other novel drug candidates have demonstrated increased cell survival by targeting the Src/phosphatase and PPARγ pathways [96,97]. Hence, although a multitude of mechanisms have been shown to circumvent destructive processes in diseased neurons, further studies are required to investigate their potential to confer disease modification in human patients.

### 4.3. Regeneration of Lost Neurons

Given that symptoms of PD manifest only after years of neuronal degeneration, a challenge for the above treatment modalities is that by the time patients are symptomatic, vast neuronal populations have already been lost [98]. Leaving aside the challenges of identifying neural degeneration before symptom onset, for those who already suffer from clinical disease, replacing lost neuronal populations may be the only hope for meaningful disease recovery. For decades, stem cell therapy has been regarded as the great hope for a cure. Dopamine progenitor cells can be derived from pluripotent fetal cells; however, the ethical quandary of harvesting fetal tissue remains a barrier to ongoing research. Recent advances in adult-derived pluripotent stem cells provide hope that stem cell research in PD may yet advance [99]. Mesenchymal stem cells are the largest and most easily accessible reservoir of adult stem cells, but research into their efficacy in PD remains limited [100]. Song et al. demonstrated that adult neural progenitor cells may be introduced to the basal ganglia through astrocyte co-grafting [101]. As of 2023, there were 13 human trials investigating the use of stem cells for the treatment of PD [102]. Stoddard-Bennet and Pera point out the inherent risk of this process, with pluripotent stem cells presenting the theoretical risk of tumors and also severe immune reactions within the host [103].

The human adult brain retains a reservoir of pluripotent neural progenitor cells that could be stimulated to regenerate the patients’ own dopaminergic neurons, thereby circumventing the need for stem cell grafting. These cells are responsive to neurotrophic molecular signals such as glial cell line-derived neurotrophic factor, fibroblast growth factor 2, and cerebral dopamine neurotrophic factor (CDNF), all of which have been shown to change concentration in PD brains [104]. Cell and animal-based trials have demonstrated the practicality of viral vectors to introduce genes expressing glial cell line-derived neurotrophic factor and neurturin into dopaminergic cells of the SNpc [105]. A phase 1 trial of direct intraputamenal injections of CDNF showed good tolerability, though a 12-month treatment of moderate to severe PD sufferers did not meet the secondary endpoint of improved PD motor scores [106]. It is still hoped that increased expression of neuronal growth factors could protect against neuronal death and promote neuronal arborisation.

Other neural regeneration signaling pathways have also shown promise. Pirodipine is a small molecule under development for the treatment of Huntington’s disease that has also been shown to stimulate dopamine neuron regeneration through activation of the sigma-1 receptor [107]. As an interesting side note, it has been postulated that deep brain stimulation, a symptomatic therapy for advanced PD, may in fact stimulate STN neuron regeneration through increasing brain-derived neurotrophic factor (BDNF) concentrations [108]. Further trials are needed to explore the potential of these and other neurotrophic factors to alter PD trajectory.

### 4.4. Repurposing Old Drugs

Repurposing available drug therapies represents the fastest and most cost-effective approach to disease modification. This paper has already discussed the potential advantages of some current immune modulators, beta-agonists, caffeine, dietary antioxidants, and endocannabinoids.

Until recently, it was posited that monoamine oxidase (MAO) inhibitors, drugs commonly used to inhibit dopamine metabolism to treat symptomatic PD, may also protect dopaminergic neurons [109]. Recent longitudinal studies, however, have not demonstrated this advantage of MAO inhibitors. On the other hand, an anti-cancer (anti-VEGF) agent, SU4312, protects against MPTP-associated neurotoxicity, at least in part, through its inhibition of MAO-B [110]. A meta-analysis from 2016 looked at the apparent advantage of anti-hypertensives to protect against PD [111]. Intriguingly, in this study, it was calcium channel blockers, rather than beta-blockers, which were most associated with reduced PD risk. Dopaminergic fibers within the basal ganglia have self-generating Ca^2+^ channels that increase their metabolic demand. Unfortunately, the Ca^2+^ channel blocker isradipine did not demonstrate a disease-modifying effect in adult participants [112]. In a recent cohort study, Vitamin B12, a critical precursor in myelin production and other nerve cell functions, did not demonstrate an advantage for PD sufferers [113]. Exanatide, a GLP-1 agonist used for diabetes management, has been shown to modify neuron survival through various signaling cascades, including microglial activity modification [114,115]. A small, well-designed double-blind trial incorporating 62 participants and a 12-week washout period failed to demonstrate a meaningful change in disease trajectory. A more recent study of exendin-4, another GLP-1 analogue, demonstrated disease-modifying effects in a PD mouse model [116]. NLY01, a brain-penetrating, long-acting version of exenatide, failed to demonstrate a disease-modifying effect in a 36-week randomized controlled trial of 255 participants with early PD [117]. Finally, it has been proposed that statin therapy may confer neuronal protection by upregulating synaptic complexity and VMAT receptor density in dopaminergic neurons, though evidence for this effect is limited [118].

Despite the suspected immune basis of neurodegeneration in PD, there has been surprisingly little focus on well-established immune modulatory drugs used for treating other neuroinflammatory conditions, particularly those used to treat multiple sclerosis (MS). Fingolimod (FTY720) is a moderate to high efficacy treatment for MS, which is a modulator of S1P activity. FTY720 treatment of 6-OHDA Parkinson’s mouse models protects against dopaminergic neuron loss [119]. Given its proven safety in humans, this is a compelling approach for future trials in PD.

**Table 1:** Preclinical trials.

Intervention/Agent	Mechanism of Action	Model of Disease	Outcome/Status	Reference
*Alpha* *-Synuclein* *Targeted*				
ABT-888	PARP-1 inhibitor	dSTR aS injected C57BL/6j mice	Suppression of aS fibrillar formation	[40]
Salbutamol	Beta-2 receptor mediated SNCA gene suppression	Norwegian population association study (4 million people)	PD incidence in regular salbutamol users vs. non-users, OR = 0.66 (CI 0.58 to 0.76)	[55]
SIFTO	m6A-dependent regulation of ATM mRNA	MPTP mouse model	Suppression of aS upregulation and reduced dopaminergic neuron death	[56]
GELDENAMYCIN	HSP70 induction	MPTP mouse model	Reduced dopaminergic neuron loss	[62]
Nanoca	Upregulation of tfEB mediated aS clearance	MPP^+^ mouse model	Reduced dopaminergic neuron loss	[64]
GYY4137	Reduced oxidative stress and reduced aS nitration	MPTP mouse model	Reduced dopaminergic neuron loss	[67]
NILOTINIB	C-abl inhibitor	MPTP mouse model	Reduced dopaminergic neuron loss	[68]
*Oxidative Stress*/*Mitochondria*				
NAC (ORAL + INTRAVENOUS)	Anti-oxidant	6-OHDA mouse model	Enhanced dopaminergic neuron viability	[82]
CELASTROL	Anti-oxidant, NrF2 pathway activation	MPTP mouse model	Enhanced dopaminergic neuron viability	[87]
AEOL11207 + AEOL11114 (METALLOPORPHYRINS)	Anti-oxidant	6-OHDA mouse model	Reduced cytokine production, reduced microglial activity and enhanceddopaminergic neuron viability	[88]
RAPAMYCIN + COENZYME Q10	Anti-oxidant	Ex vivo stem cells with LRRK2 or PINK-1 mutation	Mitochondrial protection	[89]
UFP-512	Delta-opioid receptor agonist	Ex vivo PC12 cells under oxidative stress	Upregulated PARKN mediated mitophagy	[90]
HYDROCORTISONE	PARKN upregulation	6OHDA mouse model	Enhanced dopaminergic neuron viability	[91]
*Immune Modulation*				
C-DIM12	Microglial modification	MPTP mouse model	Enhanced dopaminergic neuron viability	[94]
LYPOSOME VECTOR DELIVERY OF HYDROCORTISONE	Macrophage modificatoin	6-OHDA mouse model	Enhanced dopaminergic neuron viability	[95]
MDG548	PPAR-gamma mediated immunomodulation	MPTP mouse model	Enhanced dopaminergic neuron viability	[96]
PHLOROGLUCINOL DERIVATIVES	Src/phosphatase mediated immunomodulation	MPTP mouse model	Reduced neuroinflammation	[97]
FINGOLIMOD	S1p modulation	6-OHDA	Enhanced dopaminergic neuron viability	[119]

**Table 2:** Clinical Trials.

Intervention/Agent	Mechanism of Action	Trial Phase	Outcome	Reference
*Alpha-Synuclein*				
UCB0599	Inhibition of aS misfolding and aggregation	Phase 1/1b	Good tolerability amongst 94 volunteers	[60]
NILOTINIB	c-abl tyrosine kinase inhibitor	Phase 1	75 PD patients—well tolerated, agent detectible in CSF	[70]
VODOBATINIB	c-abl tyrosine kinase inhibitor	Phase 1	Good tolerability, superior CSF penetrance to nilotinib	[72]
PDO1A	Active immunistation against aS	Phase 1	32 participants with mild PD showed reduced CSF aS concentrations	[73]
PRASINEZUMAB	Passive immunotherapy against aS	Phases 1 and 2	316 mild PD participants—good tolerability, failed to reach primary endpoint	[75,76]
CINPANEMAB	Passive immunotherapy against aS	Phase 2	357 mild PD participants—failed to reach primary endpoint	[77]
*Oxidative Stress*/*Mitochondria*				
VITAMIN E	Termination of oxidative chain reactions	Cohort meta-analysis Phase 2 (DATATOP)	Reduced IPD incidence in vitamin E users 800 early PD participants randomised— Modest reduction in UPDRS score at 12 weeks compared with placebo	[78,80]
IV + ORAL NAC	Anti-oxidant	Phase 1	42 participants with early PD showed improved DAT binding in caudate and putamen	[83]
INTRANASAL GLUTATHIONE	Anti-oxidant	Phase 2	45 participants HY stage 1–3 PD randomised —no improvement in UPDRS from placebo over three month treatment	[84]
INOSINE	Induction of urate-mediated anti-oxidant properties	Phase 1 Phase 2 (SURE-PD3)	Increased CSF urate, well tolerated 298 participants with PD randomised—failed to reach primary endpoint	[85,86]
INTRAPUTAMENAL INJECTION OF CDNF	Neurotrophic stimulation of putamenal stem cells	Phase 1	Goold tolerability	[106]
ISRADIPINE	?blockade of Ca2+ channel induced oxidative stress	Phase 3	336 participants with early PD randomised—failed to reach primary endpoitn	[112]
NLY01 (EXENATIDE)	GLP-1 agonist -Modification of microglia	Phase 2	255 participants with early PD randomised—failed to reach primary endpoint	[117]

## 5. COVID-19: A Unique Window into the Pathogenesis of IPD?

COVID-19 is a once-in-a-century pandemic that, at the time of writing this paper, has infected well over a billion people worldwide. Acute and chronic complications of COVID-19 infection have been reported in almost every organ system, and the nervous system is no exception [120]. For these reasons, the COVID-19 pandemic presents a unique opportunity to investigate the mechanisms by which neurodegenerative diseases such as Parkinson’s Disease may arise [121].

If there is indeed a link between COVID-19 and Parkinson’s Disease, it will emerge over time as previously infected patients age [122]. If we examine the already recognized relationship between Parkinson’s Disease and viral infections, then there is indeed cause for concern. In 2009, H5N1, the ‘Avian flu’, was shown to induce inflammatory cascades and protein aggregation in mouse neural tissue mimicking the changes shown in PD, and these mice subsequently demonstrated neural degeneration specifically in the SNpc [123]. Encephalitis lethargica is an inflammatory CNS condition characterized by hypersomnolence, akinesis, and abulia that followed the emergence of the Spanish flu [124]. Patients who recovered often suffered from long-term Parkinsonism, a syndrome referred to as ‘Post-Encephalitic Parkinsonism’ (PEP), suggesting these patients’ extrapyramidal symptoms may have been a direct result of the CNS changes triggered by the H1N1 virus [125]. Indeed, studies have demonstrated that the H1N1 virus can induce CNS autoimmunity through the induction of pro-apoptotic microglia; a mechanism already thought to be involved in the genesis of PD [126]. Intriguingly, posthumous biopsies of PEP patients demonstrated tau-rich neurofibrillary tangles and an absence of Lewy bodies, indicating the underlying disease process of PEP is in some ways pathologically distinct from sporadic PD, and histologically is more reminiscent of Progressive Supranuclear Palsy [127].

Tissue biopsies in most cases of COVID-19-related neurological injury have not demonstrated direct virus infiltration of neuronal tissue. Despite this, it is now widely accepted that in rare cases, coronaviruses such as SARS-CoV-2 can also access the CNS and generate harmful downstream cell processes, though the mechanism by which they access the CNS is still not completely understood. Proposed mechanisms include direct neuron-to-neuron communication via the olfactory bulb, lymphatic spread, or direct haematogenous spread following the breakdown of the blood-brain barrier during the acute inflammatory phase The avidity of certain neuronal populations for COVID-19 invasion can be explained by the expression of cell surface ACE2 receptors, the receptors to which the COVID spike protein binds and thereby gains entry into cells [128]. Mouse models suggest COVID-19 can access the vertebrate CNS via the nasal route and take up residence in neuronal regions known to have a high density of these receptors, including the basal ganglia, brainstem, and hippocampus [129]. Of some concern, coronaviruses similar to COVID-19 have been shown to remain latent within resident CNS leucocytes and neurons for years after infection, potentially influencing cellular processes decades after acute disease recovery [130]. Alpha-synuclein has been shown to be upregulated following exposure to the West Nile virus, which some interpret as evidence that this protein is operating as a resident anti-viral defense mechanism. By extension, it could be possible that exposure to other viruses, including COVID-19, could lead to alpha-synuclein accumulation and the subsequent emergence of Parkinson’s Disease. Such a finding would lend further support to the hypothesis that alpha-synuclein is indeed pathogenic in the development of PD. On the other hand, cell models have also shown that SARS-CoV-2 (COVID-19) can induce the death of dopamine brainstem neurons through the direct activation of destructive cell processes, including the activation of caspases 2, 3, and 8, as well as the NFkappaB cascade within dopaminergic cells [131].

## 6. Conclusions and ‘Where to from Here’

Much has been learnt about the pathogenesis of Parkinson’s Disease. While the fundamental mechanism of neuron death remains debated, it is becoming clear that it is driven by a complex interplay between alpha-synuclein aggregation, reactive oxygen species, mitochondrial dysfunction, and innate immune processes. These discoveries have opened the door to a range of potential avenues to disease modification by arresting these neuron death processes. To date, no therapy has been shown to produce a profound and sustained improvement in human participants, and much more must be done to explore the potential therapeutic avenues discussed above.

One major barrier to disease-modifying treatments is that by the time patients are symptomatic, neural degeneration has been occurring for decades. More must be done to identify patients in the earliest stages of neural degeneration to introduce treatments before patients become symptomatic. Such an achievement will require more foundational knowledge of the factors predisposing patients to PD so that appropriate candidates can be identified for surveillance. Surveillance programs could monitor for the emergence of early premotor features, including anosmia, autonomic dysregulation, or REM sleep behaviour disorder. Of these, RBD has emerged as the frontrunner, as it demonstrates a high degree of specificity for conversion to a synucleinopathy (PD, MSA, DLB, or PDD), with with 80% of patients developing a synucleinopathy within ten years of the emergence of dream enactment behaviours during REM sleep [132]. The link between RBD and PD is likely an underlying failure of REM sleep atonia, which is challenging to identify in the absence of frequent, typical RBD-related sleep movements and in patients without a bed partner. For this reason, RBD lacks the required sensitivity of a prodromal disease marker.

Research into prodromal disease biomarkers has intensified recently with the discovery of real-time quaking-induced conversion (RT-QuiC) as a validated method of detecting aS in many different tissues, including CSF, serum, and skin [133,134]. Subsequent studies in RBD participants validate RT-QUiC as a method of prodromal disease detection, providing hope that a simple blood, skin, or other non-invasive tissue sample could be used for widespread screening of prodromal PD and related synucleinopathies [135]. Another more costly approach could utilize either VMAT PET or SPECT imaging to look for early reduction in dopamine activity, though again, by the time such changes are observed, much neuronal degeneration has likely already occurred. Furthermore, up to 20% of patients with clinical PD do not demonstrate evidence of dopamine deficiency on imaging (SWEDD, ‘scans without evidence of dopamine deficiency’).

Many of the candidate interventions discussed in this review were first tried on mouse models of PD, such as the MPTP model. MPTP is especially toxic to dopaminergic neurons, thereby mimicking many of the motor features of PD. However, direct toxicity of dopaminergic neurons does not reflect the physiological processes that cause the loss of dopaminergic neurons in human PD, nor does it replicate the extra-dopaminergic changes demonstrated in humans [136]. It is, therefore, of little surprise that disease-modifying effects in animal models have not been replicated in human subjects.

Another major challenge to disease modification research in human trials relates to study design. There is currently no agreed-upon objective measure of neuronal loss, so treatment efficacy relies on subjective measures of clinical status such as the UPDRS Parkinson’s scale. Further to this, most phase 2 trials are typically no more than six months in duration. PD moves slowly, with often times an almost imperceptible progression in motor impairment over any six-month period. For this reason, demonstrating a separation in outcomes between control and treatment arms over this short time is inherently problematic. Apart from the problem of inter- and intra-rater reliability that such scales introduce, these scales are unable to differentiate between a treatment’s symptomatic benefit and true disease modification. Studies have attempted to get around this issue by introducing a clinical ‘washout period’ prior to clinical assessment, but this adds significantly to the study duration, and there is currently no way of knowing how long such washout periods should be. The consensus opinion remains that the UPDRS Parkinson’s scale is the gold standard for assessing motor outcomes in long-term disease-modifying trials, though experts now advocate the inclusion of non-motor parameters to provide a broader measure of PD severity and progression [137,138]. The identification of a clinical biomarker of active neural degeneration would represent an elegant solution to these limitations [139]. Trials on CSF aS levels have provided confounding results with lower CSF aS modestly correlating with the risk of motoric PD [140]. McGhee et al. provide a thorough review of recent clinical trials of disease modification and argue that the current gold standard trial design is a long-term follow-up study aiming to demonstrate a sustained and robust divergence in clinical outcome measures between cases and controls [141].

When we consider the above challenges, it could be argued that the greatest hope of a cure for sporadic PD lies in neural regeneration utilizing stem cell therapy. Such therapies could theoretically reverse nerve cell degeneration, leading to a rapid and sustained clinical improvement that does not rely on early detection of symptoms or protracted studies looking for improved clinical outcomes over decades. Grafted stem cells carry the theoretical risk of tumors and host rejection, so a focus on neurotrophic factors to stimulate autologous neural stem cells presents a compelling approach.

As global populations age, neurodegenerative diseases are emerging as one of the major drivers of human suffering and healthcare burden. For sporadic PD, dopamine-based therapies eventually lose efficacy, and patients suffer from mounting non-motor features that significantly contribute to patient morbidity and mortality. For these reasons, there is a critical urgency for further research into disease modification in PD. The limited success of past efforts is sobering. On the other hand, the diversity and international reach of current efforts to discover new therapeutics provide much hope for the future.

## Data Availability

No new data were created or analyzed in this study.

## Abbreviations

ACE2	Angiotensin converting enzyme 2	MPTP	Methyl-phenyl-tetrahydro-pyridine
AD	Alzheimer’s disease	mRNA	messenger ribose nucleic acid
ADP	Adenosine diphosphate	MSA	Multisystem atrophy
AIF1	Apoptosis-inducing factor	mtPTP	Mitochondrial permeability transition pore
aS	alpha-synuclein	NAC	N-acetyl cysteine
BAX	BCL2-associated X protein	NLR	Neutrophil-to-lymphocyte ratio
BDNF	Brain derived neurotrophic factor	Nrf2	Nuclear factor erythroid 2-related factor 2
Ca^2+^	Calcium cations	PARP	Poly ADP-ribose polymerase
c-abl	cellular Abelson murine leukaemia	PC12	Phaechromocytoma cell line
	viral oncogene homolog 1	PD	Parkinson’s Disease
CBD	Cannabidiol	PDD	Parkinson’s Disease Dementia
CDNF	Cerebral dopamine neurotrophic factor	PEP	Post-encephalitic parkinsonism
Chr	Chromosome	PET	Positron emission tomography
CNS	Central nervous system	PPR	Peroxisome proliferator-activated receptor
COVID	Coronavirus disease	REM	Rapid eye movement
CSF	cerebrospinal fluid	ROS	reactive oxygen species
Cyt c	Cytochrome C	RT-QuiC	Real-time quaking-induced conversion
DAT	Dopamine transporter	SNCA/SCNA	synuclein alpha
DLB	Dementia with lewy bodies	SNpc	Substantia nigra pars compacta
Fe^2+^/^3+^	Iron cations	SPECT	Single photon emission computed tomography
GBA/GCA	Glucocerebrosidase	SWEDD	Scans without evidence of dopamine deficiency
GLB	Glucagon-like peptide	S1P	Sphingosine-1-phosphate
HAT	Histone acetyltransferase	S129	Serine 129
HDAC	Histone deacetylatse	THC	etrahydrocannabinoid
HSF-1	Heat shock factor 1	TLR	Toll like receptor
Hsp	Heat shock protein	TNF	Tumour necrosis factor
H2S	Hydrogen sulfide	VEGF	Vascular endothelial growth factor
IFN	interferon	VMAT	Vesicular monoamine transporter
IL	Interleukin	VPS35	Vacuolar Protein Sorting 35
LRRK2	Leucine-rich repeat kinase 2	6-OHDA	6-hydroxydopamine
MAO	Monoamine oxidase		
MAPT	Microtubule-associated protein tau		
MDS-UPDRS	Movement disorders society-unified Parkinson’s disease rating scale		
MPP	methyl-phenyl-pyridinium		
